# Multiple Pulp Stones in Primary and Developing Permanent Dentition: A Report of 4 Cases

**DOI:** 10.1155/2012/408045

**Published:** 2012-08-28

**Authors:** Mohita Marwaha, Radhika Chopra, Payal Chaudhuri, Atul Gupta, Jayna Sachdev

**Affiliations:** Department of Pedodontics & Preventive Dentistry, SGT Dental College, Hospital & Research Institute, Budhera 123505, India

## Abstract

Pulp stones are foci of calcification or discrete calcifications in the dental pulp. They are frequently found on bitewing and periapical radiographs, but their occurrence in entire dentition is unusual. We are reporting four cases in which the occurrence of pulp stones ranged from their presence in just primary teeth (Cases 1 and 2) to involvement of young permanent teeth also (Case 3) and even unerupted permanent teeth (Case 4). In all the cases, dental, medical, and family histories as well as the findings from the clinical examination of the patient were not contributory. Histopathological report revealed true denticle. Metabolic evaluation of patients through liver function test, kidney function test, and blood investigation did not show any metabolic disorders. Patients were also evaluated for any systemic, syndromic, or genetic involvement, but this was also noncontributing. Therefore, it is suggested that these unusual cases may be of idiopathic origin.

## 1. Introduction

Pulp calcifications occurring throughout dentition are uncommon and are usually associated with systemic or genetic diseases such as dentine dysplasia, and dentinogenesis imperfecta [1]. Free pulp stones are found coronally within the pulp tissue and are most commonly seen on radiographs [2]. Pulp stones extending to the entire primary dentition are also infrequent. The purpose of this paper is to present four cases: first with pulp stones in all primary molars with taurodontism, second with pulp calcifications in all primary molars, third with pulp stones in all the primary molars, mandibular permanent central incisors, and first molars, and fourth, with pulp stones in all primary molars, permanent first molars and also in developing unerupted permanent mandibular second molars, permanent mandibular canines, and first premolars. The present case report depicts presence of pulp stones, without any metabolic disturbances and syndrome, which may be suggestive of its idiopathic origin.

**Case 1**

**Figure 1:**
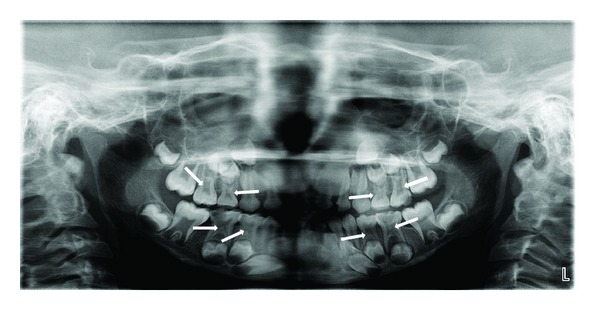
OPG revealing pulp stones and taurodontism in all primary molars (Case  1).

**Figure 2:**
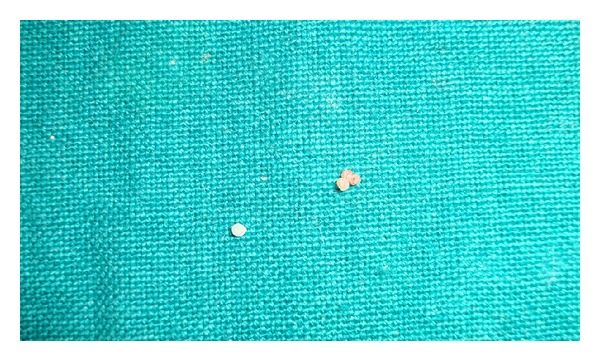
Pulp Stones.

A 6-year-old girl reported to the clinic with the chief complaint of decayed left posterior teeth since the last three months. Her family/medical history was noncontributory. Intraoral examination revealed several decayed teeth including all the primary molars. So, an OPG was advised which showed taurodontism and pulp stones in all primary molars (Figure 1). Pulpectomy was performed in primary maxillary and mandibular left second molars, and the pulp stones removed from the chamber (Figure 2) were sent for histopathological examination and were found to be true denticle. Primary mandibular right second molar was extracted followed by band and loop space maintainer. The rest of the decayed teeth were restored with glass ionomers cement.

**Case 2**

**Figure 3:**
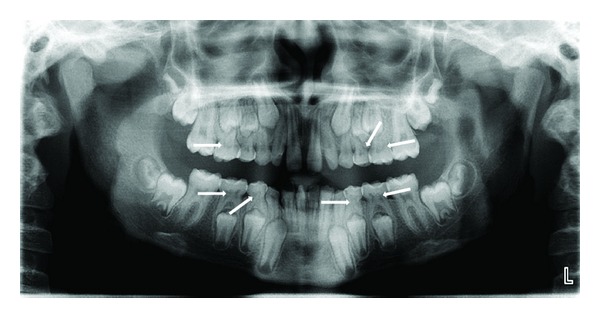
OPG revealing pulp stones in all primary molars (Case  2).

A 12-year-old male patient reported to clinic with chief complaint of malaligned teeth in lower front region. Intraoral examination revealed retained primary mandibular incisors. The Panoramic radiograph revealed pulp stones in all primary molars (Figure 3). So, planned treatment was extraction of retained primary mandibular incisors followed by fixed orthodontic therapy. As there was no carious involvement of primary molars, they were left untreated.

**Case**

**Figure 4:**
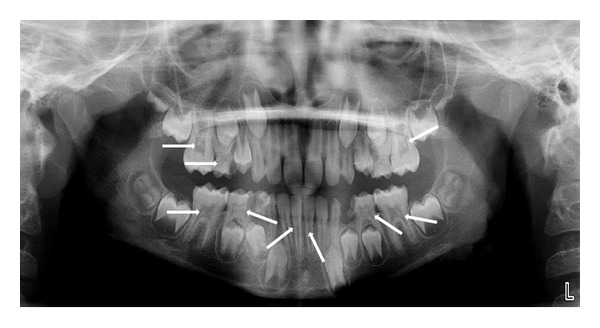
OPG revealing pulp stones in all second deciduous molars, first permanent molars, and mandibular central incisors.

A 10-year-old male patient reported with the chief complaint of decayed right posterior teeth for 2 months. The family and medical history was noncontributory. Intraoral examination revealed deep carious lesions in many teeth. The Panoramic radiograph revealed pulp stones in all second primary molars, permanent first molars, and mandibular central incisors (Figure 4). So, planned treatment was glass ionomer restorations in all decayed teeth; indirect pulp capping was performed in primary maxillary right second molar and extraction of primary mandibular right and left first molars; the remaining asymptomatic teeth were left untreated.

**Case**

**Figure 5:**
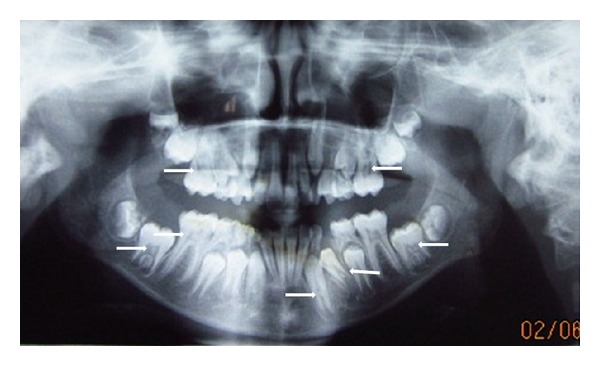
OPG revealing pulp stones in all primary molars and almost in all permanent teeth.

An 11-year-old male patient reported with the chief complaint of decayed left posterior teeth for 1 month. Intraoral examination revealed carious lesions in many teeth. The Panoramic radiograph revealed pulp stones in all primary molars and permanent first molars. Also calcifications were observed in developing unerupted permanent mandibular second molars, permanent mandibular canines, and first premolars (Figure 5). So, planned treatment was restorations in all decayed teeth and extraction of primary mandibular right first molar followed by lingual arch.

## 2. Discussion

Pulp stones are foci of calcification or discrete calcifications in the dental pulp. They may exist freely within the pulpal tissue or embedded/attached to dentine [3]. Stones may occur as a single mass or as several small radio-opacities within pulp chambers or may exist into root canals [4]. A single tooth may have from 1 to 12 stones or even more, with sizes varying from minute particles to large masses [3]. They occur in all tooth types but occur most commonly in molars [2]. 

Pulp stones can be structurally classified based on location [5]. Structurally, they can be true and false pulp stones. True stones are made up of dentine and lined by odontoblasts, whereas false pulp stones are formed from degenerating cells of the pulp that are mineralized. A third type, “diffuse” or “amorphous” pulp stones, is more irregular in shape [6]. Based on the location, they can be embedded, adherent, and free [5]. Embedded stones are found most frequently in the apical portion of the root, and they are more attached to dentine as compared to adherent stones. Adherent stones are attached to the wall of pulp space, but they are not fully enclosed by dentine. Both adherent and embedded pulp stones can interfere with root canal treatment if they cause occlusion of the canals.

Free pulp stones are found coronally within the pulp tissue and are the most commonly seen on radiographs. They are very common and vary in size from 50 um in diameter to several millimetres where they may occlude the entire pulp chamber [7]. Pulp stones vary in size, ranging from microscopic particles to larger masses that almost obliterate the pulp chamber with only the large masses being radiographically apparent. In all our cases, radiograph examination revealed large pulp stones located in the pulp chamber. 

Sayegh and Reed [7] reported that the incidence of calcification in carious teeth from children and young adults (10–34 years old) was nearly 5 times greater than that in noncarious teeth. This supports the theory that pulp calcification is, under normal condition, a physiological process. Under pathological conditions (like caries), the process may speed up. The influence of caries on pulp stone formation may actually be related to properties of dentine such as the number and dimensions of tubules, and the progression rate, and activity of the disease. These would influence the rate of bacterial toxin penetration. In our cases, although many of the teeth were carious, noncarious teeth and even unerupted teeth had pulp stones suggesting idiopathic aetiology rather than pathological one. 

Kumar et al. [8] conducted a study in 120 primary maxillary and mandibular extracted teeth, evaluated them radiographically, and concluded 25% of second molars presented evidence of pulp calcifications and approximately 3% of central incisors were calcified. The low occurrence of pulp calcifications in primary teeth supported the view that the occurrence of pulp calcification increases with age. However, Arys et al. [4] found that age did not have any influence on the occurrence of pulp calcifications. Their study consisted of 42 primary molars with less than one-third of their root resorbed. Forty-two healthy children of both sexes were selected, aged between 5 and 13 yrs. The teeth were examined by microradiography and light microscopy, and results revealed that pulp stones were present in 78% of the molars, with 95% of the material showing some form of pulp calcification. There was lower incidence of pulp stones in treated and carious molars.

Pulp calcification also occurs as sequelae to trauma to the primary dentition [9]. In cases with repeated traumatic injuries, the chances of pulp calcification are doubled compared to single trauma. It is a common finding associated with the healing process following traumatic injuries. Prevalence of pulp calcification in injured primary teeth that were diagnosed by radiographs varied from 6.1% to 35.9%.

Yaacob and Hamid [10] reported that free or attached pulp stones were the most common type of calcification. They selected 120 teeth of children aged between 3 and 11 yrs, examined them histologically, and reported 6.7% of prevalence of pulp stones.

In Cases  1 and  2, pulp stones were noticed along with taurodontism in primary molars. The term taurodontism was first introduced by Sir Arthur Keith in 1913 [11]. The taurodontic teeth are identified by elongated pulp chambers and apical displacement of bifurcation or trifurcation of the roots. Etiology of taurodontism is diverse commonly attributed to the failure of invagination of the epithelial root sheath sufficiently early to form the cynodont. Autosomal transmission of the trait has also been observed.Taurodontism can occur alone limited to one or more teeth or it can be associated with various syndromes like Down's syndrome, Klinefelter's syndrome, and so forth [12, 13]. Taurodontism may be unilateral or bilateral and affects permanent teeth more frequently than primary teeth. Taurodontism may be classified as mild, moderate, and severe (hypo, meso, and hyper, resp.) based on the degree of apical displacement of the pulpal floor [14, 15].

So far, only 2 cases have been reported by Kosinski et al. [14] of pulp stones associated with taurodontism. They demonstrated short, conical, and misshapen roots with pulp stones in the pulp chamber of taurodontic teeth. Mandibular molars are found to be affected more than maxillary molars [16].The similar findings were recorded in Case 1. As a taurodont shows wide variation in size and shape of pulp chamber with varying degrees of obliteration and canal configuration, root canal therapy becomes a challenge. In Case  4, calcifications were also observed in unerupted developing permanent teeth and till date to the best of our knowledge no case has been reported of the same findings. 

In our present cases, the pulp stones were found in young patients, which is contrary to the general concept of pulp stone formation usually seen in older age group or in association with certain syndrome. In our cases, no correlation could be established between pulp stones and any genetic, systemic, or metabolic findings. However, the same findings were reported by Siskos and Georgopoulou [17], Bahetwar and Pandey [18], and Donta et al. [19]. Thus, it may be suggested that these stones were of idiopathic origin. Further studies are required to evaluate the exact mechanism and aetiology of pulp calcification which would be able to clarify the fact that generalized pulp calcification is not merely an age-changed phenomenon attributed for this condition.
